# Ensembling Variable Selectors by Stability Selection for the Cox Model

**DOI:** 10.1155/2017/2747431

**Published:** 2017-11-15

**Authors:** Qing-Yan Yin, Jun-Li Li, Chun-Xia Zhang

**Affiliations:** ^1^School of Science, Xi'an University of Architecture and Technology, Xi'an, Shaanxi 710055, China; ^2^School of Mathematics and Statistics, Xi'an Jiaotong University, Xi'an, Shaanxi 710049, China

## Abstract

As a pivotal tool to build interpretive models, variable selection plays an increasingly important role in high-dimensional data analysis. In recent years, variable selection ensembles (VSEs) have gained much interest due to their many advantages. Stability selection (Meinshausen and Bühlmann, 2010), a VSE technique based on subsampling in combination with a base algorithm like lasso, is an effective method to control false discovery rate (FDR) and to improve selection accuracy in linear regression models. By adopting lasso as a base learner, we attempt to extend stability selection to handle variable selection problems in a Cox model. According to our experience, it is crucial to set the regularization region Λ in lasso and the parameter *λ*_min_ properly so that stability selection can work well. To the best of our knowledge, however, there is no literature addressing this problem in an explicit way. Therefore, we first provide a detailed procedure to specify Λ and *λ*_min_. Then, some simulated and real-world data with various censoring rates are used to examine how well stability selection performs. It is also compared with several other variable selection approaches. Experimental results demonstrate that it achieves better or competitive performance in comparison with several other popular techniques.

## 1. Introduction

Variable selection is a classical problem in statistics and has enjoyed increased attention in recent years due to a massive growth of high-dimensional data across many scientific disciplines. In modern statistical applications, the number of variables or covariates *p* often exceeds the number of observations *n*. In such settings, the true model is often assumed to be sparse, in the sense that only a small proportion of the *p* variables actually relates to the response. Thus, variable selection is fundamentally important in statistical analysis of high-dimensional data. With a proper selection method and under suitable conditions, we are able to build a good model to interpret the relationship between covariates and our interested outcome more easily, to avoid overfitting in prediction and estimation, and to identify important variables for applications or further study.

For variable selection, many researchers focus on multiple linear regression models. To emphasize that variable selection methods are useful for other statistical models as well, we use a different statistical model, that is, a Cox's proportional hazards model (abbreviated as Cox model) [1], as the platform in this context. The Cox model was first proposed for exploring the relationship between the survival of a patient and some explanatory variables. As a matter of fact, the Cox model [2, 3] nowadays is one of the most commonly used forms in semiparametric models and it can not only solve the issues of censored data, but also analyze the influence of various factors on survival time simultaneously. A brief mathematical description of the Cox model is given as follows.

Suppose that there are *n* observations {(*y*_*i*_, **x**_*i*_, *δ*_*i*_)}_*i*=1_^*n*^ of survival data. For an individual *i*, *y*_*i*_ denotes its survival time and **x**_*i*_ = (*x*_*i*1_, *x*_*i*2_,…,*x*_*ip*_)^*T*^ stands for the observed data for the *p* covariates. At the same time, *δ*_*i*_ ∈ {0,1} is a censoring indicator variable, where *δ*_*i*_ = 0 means that *y*_*i*_ is right-censored. Let *h*(*t*) be the hazard rate at time *t*; the generic form of a Cox's proportional hazards model can be expressed as

(1) $$\begin{array}{c} h\left(\;t\;|\;x\;\right)=h_{0}\left(\;t\;\right)\exp\left(\;x^{T}\beta \;\right), \end{array}$$

where **β** = (*β*_1_, *β*_2_,…, *β*_*p*_)^*T*^ is a *p*-dimensional unknown coefficient vector and *h*_0_(*t*) is the baseline hazard function, that is, the hazard function at time *t* when all covariates take value zero. In general, **β** can be estimated by maximizing partial likelihood function. For convenience, we assume *h*_0_(*t*) = 1 below.

Like linear regression models, traditional methods such as subset selection [4, 5], forward selection, backward elimination, and a combination of both are among the most common methods for selecting variables in a Cox model. However, these methods will have difficulty in computation when faced with high-dimensional data. Therefore, some other methods have been proposed to overcome this problem. After lasso (least absolute shrinkage and selection operator) [6] was first proposed for linear regression models, Tibshirani [7] extended it to the Cox model. Later on, many scholars [2, 3, 8–12] developed some penalized shrinkage techniques like SCAD [13] and adaptive lasso [14] specially for Cox models.

Although the above-mentioned variable selection methods have been shown to be successful in theoretical properties and numerous experiments, their performance strongly depends on the proper setup of the tuning parameter. On the other hand, these approaches may be unstable (especially in the situation of high-dimensional data). Breiman [15] proved that uncertainty can lead to more prediction loss. What is more important, small changes in data can result in that the same method selects different models. This makes the subsequent interpretation difficult and unreliable. In order to obtain more stable, accurate, and reliable variable selection results, ensemble learning [16, 17] is one kind of extremely potential technologies.

As a hot research topic in machine learning, ensemble learning is used more and more widely in many fields of natural science and social science in last two decades. The powerful advantages of ensemble learning lie in improving the generalization capacity and enhancing robustness in the process of learning. Its main idea is to obtain a number of different base learning machines by running some simple learning algorithm and then combine these base machines into an ensemble learning machine in some way. Generally, the base learning machines should have strong generalization capability on one side, and they should also complement each other on the other hand.

The ensemble approach for statistical modeling was first proposed for solving prediction problems, aiming to maximize* prediction accuracy*. Inspired by this idea, Zhu and Chipman [18] applied bagging ensemble approach to handle variable selection problems, aiming at maximizing* selection accuracy*. Meanwhile, they pointed out that there is much difference between “prediction ensembles” (PEs) and “variable selection ensembles” (VSEs). More recently, ensemble learning methods have attracted more attention on coping with variable selection problems since they can greatly improve the selection accuracy and lessen the risk to falsely select unimportant variables and simultaneously overcome the instability of traditional methods in the high-dimensional data analysis. Because of these benefits, there are more and more researches applying ensemble learning to variable selection and putting forward some novel approaches. As far as we know, existing VSE techniques mainly include PGA (parallel genetic algorithm) [18], stability selection [19], BSS (bagged stepwise search) [20], random lasso [21], ST2E (stochastic stepwise ensemble) [22], TCSL (tilted correlation screening learning) [23], RMSA (random splitting model averaging) [24], SCCE (stochastic correlation coefficient ensemble) [25], and PST2E (pruned stochastic stepwise ensemble) [26]. It is noteworthy that these algorithms are mainly designed for handling variable selection problems in linear regression models. Only Zhu and Fan [20] investigated the performance of BSS and PGA in the Cox model.

Through analyzing these VSE techniques, it can be found that their success primarily lies in producing multiple importance measures for each predictor. By simply averaging these measures across multiple trials, the noise variables can be more reliably distinguished from the informative ones. In this process, the strength to select important variables and the diversity between the importance measures need to be preserved simultaneously [20, 22]. Stability selection applies subsampling (or bootstrap) to a selection method like lasso to improve its performance. In fact, it is an extremely general ensemble learning technique for identifying important variables. Due to the characteristics of lasso, it is very efficient in high-dimensional situations. Another good property of stability selection is that it provides an effective way to control false discovery rate (FDR) in finite sample cases provided that its tuning parameters are set properly. Due to its versatility and flexibility, stability selection has been successfully applied in many domains such as gene expression analysis [24, 27–29]. Nevertheless, we have not found any literature about applying stability selection to a Cox model. Therefore, in this paper we would like to extend it to the situation of Cox models. At the same time, we also discuss how to set appropriate values for the involved parameters so that stability selection achieves its best performance.

The remainder of the paper is described as follows. In Section 2, the details for applying stability selection to the Cox model are described. We also provide an explicit way to set its involved parameters. In Section 3, some numerical experiments were conducted to study the impact of *λ*_min_ on the behavior of stability selection and to compare its performance with other variable selection approaches for the Cox model. In Section 4, some real examples are analyzed to further study the effectiveness of stability selection. Finally, some conclusions are offered in Section 5.

## 2. Stability Selection Algorithm for the Cox Model

In this paper, we consider stability selection with lasso as its base learner. Lasso [6] is one of the most effective techniques to deal with high-dimensional linear regression problems with *p* > *n*. With respect to its application in Cox models, the core idea is to maximize the partial likelihood minus the *L*_1_ penalty function. For convenience, suppose that there are *m* unique failure times, say, *t*_1_ < *t*_2_ < ⋯<*t*_*m*_, among the *n* observations {(*y*_*i*_, **x**_*i*_, *δ*_*i*_)}_*i*=1_^*n*^. Let *j*(*i*) denote the index of the observation failing at time *t*_*i*_. The lasso algorithm needs to maximize

(2) $$\begin{array}{c} L\left(\;\beta \;\right)=\overset{m}{\underset{i=1}{\prod }}\frac{\exp\left(x_{j\left(i\right)}^{T}\beta \right)}{\sum_{j\in R_{i}}\exp\left(x_{j}^{T}\beta \right)}, \end{array}$$

under the constraint ∑_*j*=1_^*p*^|*β*_*j*_| ≤ *s*. In (2), *R*_*i*_ is the set of indices, *j*, with *y*_*j*_ ≥ *t*_*i*_ (i.e., the observations are at risk at time *t*_*i*_). Equivalently, the estimate of **β** can be obtained as

(3) β^=arg⁡maxβ∑i=1mxjiTβ−log⁡∑j∈Riexp⁡xjTβ−λ∑j=1pβj,

where *λ* is the regularization parameter which controls the trade-off between the model fitting and the coefficient shrinkage degree. At present, there are several efficient algorithms [7, 30] (such as cyclical coordinate descent) to get $\hat{\beta }$ in (3). We refer readers to the related literature for more details about the optimization strategy.

In applications, we need to first set a sensible region, say, Λ = [*λ*_lower_, *λ*_upper_], for the regularization parameter *λ* in lasso. Notice that lasso will choose* all* variables (i.e., full model) for *λ* ≤ *λ*_lower_ while choosing* none* of the variables (i.e., null model) for *λ* ≥ *λ*_upper_. By taking *K* candidate values in Λ, that is, *λ*_lower_ = *λ*_1_ < *λ*_2_ < ⋯<*λ*_*K*_ = *λ*_upper_, lasso generally employs 5-fold or 10-fold cross-validation to select an optimal value of *λ*, say *λ*_opt_. Then, the variables which have nonzero coefficient estimation under *λ*_opt_ are determined as important variables. Although lasso with *λ*_opt_ being specified in this way has good prediction performance, much evidence [14, 19, 21] has shown that it tends to choose more variables than necessary (i.e., higher FDR).

To eliminate this drawback of lasso, Meinshausen and Bühlmann [19] developed stability selection which works by choosing variables whose* selection probabilities* are large as important ones. In reality, the selection probability can be estimated by running lasso on multiple different sets. These sets can be obtained via subsampling from the given set. Specifically, stability selection first estimates the probability that variable *X*_*j*_  (*j* = 1,2,…, *p*) is important for each regularization parameter *λ*_1_,…, *λ*_*K*_, and then takes the maximum probability over Λ = {*λ*_1_, *λ*_2_,…, *λ*_*K*_} as the important measure for *X*_*j*_. Eventually, it selects important variables by a preset threshold *π*_thr_. The detailed steps of stability selection algorithm for the Cox model are listed in Algorithm 1.

As argued by Meishausen and Bühlmann [19], the prominent advantage of stability selection is to control FDR under finite sample size and simultaneously to weaken the theoretical assumptions that are required to achieve variable selection consistency (i.e., the probability that the fitted model includes only truly important variables is tending to one when *n* → *∞*). Let *V* be the number of falsely selected variables with stability selection; Meinshausen and Bühlmann [19] have proved that, under some mild assumptions, for arbitrary *π*_thr_ ∈ (1/2, 1), the expectation of *V* satisfies

(4) $$\begin{array}{c} E\left(\;V\;\right)\leq \frac{1}{2\pi_{thr}-1}\cdot \frac{q_{\Lambda }^{2}}{p}, \end{array}$$

where *q*_Λ_ represents the average number of variables selected by base learner. Roughly speaking, we can set any two parameters of *q*_Λ_, *π*_thr_, and *E*(*V*) and determine the remaining one according to the above inequality. For example, let *E*(*V*) ≤ 4 and *π*_thr_ = 0.7; then *q*_Λ_ can be specified as *q*_Λ_ = ⌈(1.6*p*)^1/2^⌉ in which ⌈*A*⌉ denotes taking the smallest integer larger than or equal to *A*. As stated in [19], *π*_thr_ is recommended to take value in the range of *π*_thr_ ∈ [0.6,0.9] and the results tend to be similar. As far as *E*(*V*) is concerned, it can be set by users according to the level of FDR that they would like to control. In general, small *E*(*V*) means to control FDR strictly so that less noise variables are falsely included. Nevertheless, too small *E*(*V*) may cause some truly important variables omitting in the final model. On the other hand, *E*(*V*) can be larger if one can accept a little higher FDR to make sure that all important variables can be included. Regarding *q*_Λ_, it should be no less than the number of truly important variables. Because we have no means to know the number of truly important variables in advance, however, one can first specify *E*(*V*) and *π*_thr_ and let *q*_Λ_ be determined automatically.

As mentioned earlier, the crucial role of stability selection is to reduce the FDR of lasso (i.e., to exclude noise variables more reliably). Intuitively, it is still difficult to identify the true sparse model if too much noise variables are falsely included every time. Thus, a minimum value of *λ* (or *λ*_min_) needs to be specified for stability selection so that every time at most *q*_Λ_ variables are chosen when *λ* ≥ *λ*_min_. Subsequently, only the *λ*'s lying in the interval [*λ*_min_, *λ*_upper_] are taken as candidate values of *λ* to implement lasso in each trial.

According to our experience, the setting of *λ*_min_ as well as Λ is crucial to the success of stability selection. However, we cannot find any detailed instruction in related literature [19, 27, 28] about how to set them. Moreover, all the existing literature related to stability selection has not discussed how to apply it in Cox models. Here, we would like to provide an explicit way to cope with this problem in the framework of Cox models. According to the proposal in [30], we can first set *λ*_upper_ for lasso in a Cox model as

(5) $$\begin{array}{c} \lambda_{upper}=\underset{1\leq j\leq p}{\max}\frac{1}{n}\overset{n}{\underset{k=1}{\sum }}\omega_{k}x_{kj}z_{k}, \end{array}$$

in which

(6) ωk=∑i∈Cksi−1si2,si=∑j=1nIyj>ti,  Ck=i ∣ yk>ti,  i=1,2,…,n,zk=1ωkδk−∑i∈Ck1si.

Here, *s*_*i*_ is the number of subjects (observations) at risk at time *t*_*i*_ and *C*_*k*_ is the set of indices, *i*, with *t*_*i*_ < *y*_*k*_ (i.e., the times for which observation *k* is still at risk). Subsequently, we can set *λ*_lower_ = *ϵλ*_upper_ with *ϵ* = 0.05 for *n* < *p* and *ϵ* = 0.0001 for *n* ≥ *p*. In order to create *K* + 1 candidate values for *λ* ∈ [*λ*_lower_, *λ*_upper_], we can set *λ*_*j*_ = *λ*_upper_(*λ*_lower_/*λ*_upper_)^*j*/*K*^ for *j* = 0,…, *K*.

Next, the parameter *λ*_min_ in stability selection can be determined by

(7) λmin=arg⁡maxλλupper−λ:λlower≤λ≤λupper,q^λ,λupper=qΛ.

Equation (7) implies that *λ*_min_ must be chosen to ensure that lasso selects at most *q*_Λ_ variables for each *λ* ∈ Λ = [*λ*_min_, *λ*_upper_]. Specifically, one can begin with *λ* = *λ*_upper_ and decrease *λ* gradually until lasso detects *q*_Λ_ variables as important (i.e., *q*_Λ_ variables having nonzero coefficients). The value of *λ* obtained at this point is exactly *λ*_min_ defined in (7). Then, only the candidate values lying in [*λ*_min_, *λ*_upper_] are considered as the candidate values for *λ* in lasso to execute variable selection.

## 3. Experimental Studies

With simulated data, some experiments are conducted in this section to investigate the impact of *λ*_min_ on the behavior of stability selection in a Cox model and to compare it with several other variable selection approaches. In order to maintain consistency and comparability, we set ensemble size *B* as 200. Each simulation was run 100 times to estimate the evaluation of a method. To simplify notations, we abbreviated stability selection as StabSel. Regarding lasso, we made use of 10-fold cross-validation to determine its optimal regularization parameter.

### 3.1. Simulation 1: Influence of *λ*_min_

Meinshausen and Bühlmann [19] stated that the threshold value *π*_thr_ is a tuning parameter whose influence is small as long as it is in the range of (0.6,0.9). According to our experience, *λ*_min_ has more significant effect in comparison with the parameter *π*_thr_. When *V* and *π*_thr_ are fixed, small *λ*_min_ will make lasso select more variables in each path. As a result, some noise variables may be falsely considered as important ones (i.e., high false positive rate). On the other hand, the noise variables can be safely filtered out by setting a large *λ*_min_. However, this may lead us to miss some signal variables (i.e., high false negative rate). Thus, *λ*_min_ plays a role in controlling the trade-off between false positive rate and false negative rate of StabSel. Due to this consideration, we fixed *π*_thr_ = 0.6 and report results for several values of *λ*_min_ in the first experiment.

Suppose that there are *p* = 8 variables, **x**_1_, **x**_2_,…, **x**_8_, with each generated from the standard normal distribution *N*(0,1). Furthermore, the variables are correlated with *ρ*(*x*_*i*_, *x*_*j*_) = 0.5^|*i* − *j*|^ for all *i* ≠ *j*  (*i*, *j* = 1,…, 8). The response **y** was generated from an exponential distribution whose hazard function is

(8) $$\begin{array}{c} h\left(\;t\;|\;x\;\right)=h_{0}\left(\;t\;\right)\exp\left(\;x^{T}\beta \;\right), \end{array}$$

where the true coefficient vector **β** = (3,1.5,0, 0,2, 0,0, 0)^*T*^. Clearly, only three variables **x**_1_, **x**_2_, **x**_5_ are truly important and the remaining ones are unimportant. We took *n* = 50 and conducted three experiments with censoring rates 0%, 20%, and 40%, respectively. For the censoring mechanism, a censoring time *t*_*i*_ is generated independently and uniformly from [0, *η*] for each observation. If *y*_*i*_ > *t*_*i*_, we replaced *y*_*i*_ with *t*_*i*_ and then let *δ*_*i*_ = 0. Here, the parameter *η* was chosen to achieve some desired censoring rates. For example, *η* = 45 corresponds to 20% censoring rate and *η* = 4 corresponds to 40% censoring rate. Aiming at evaluating the performance of StabSel for a given *λ*_min_, we computed the* selection frequency* of StabSel in each case. Specifically, the selection frequency was calculated as, among 100 simulations, the minimum, median, and maximum number of times that the important and unimportant variables (*IV*  and  *UIV*) are selected by StabSel, respectively. Interested readers can refer to [26] for the detailed definition of selection frequency.  Table 1 summarizes the results for the cases with different centering rates.

The results in Table 1 demonstrate that StabSel using a relatively large *λ*_min_ performs slightly better in excluding unimportant variables. However, the side effect is that it more likely misses some truly important variables. In other words, StabSel controls false discovery rate (or false positive rate) quite effectively with a relatively large *λ*_min_, but this will cause it to behave poorly in terms of catching important variables. To improve its selection accuracy, we must reduce *λ*_min_. Nevertheless, this inevitably allows more false discoveries. In practice, it is worthy of choosing an appropriate value for *λ*_min_ depending on whether our emphasis is more on false positive rate or false negative rate. Moreover, we need to pay more attention to the tuning of *λ*_min_ if the censoring rate is high.

### 3.2. Simulation 2: Performance Comparison on a Cox Model with High-Dimensional Data

In this subsection, we concentrated on applying StabSel and lasso to a Cox model with high-dimensional data. To generate the design matrix, the following two simulated datasets were generated by following the strategy in [19].

**Case 1 .**

**x**_*k*_ ~ *N*(0, **I**_*n*_), where *k* = 1,2,…, *p* and *p* = 1000, *n* = 100.

**Case 2 .**

**x**_*k*_ = *f*_*k*,1_*ϕ*_1_ + *f*_*k*,2_*ϕ*_2_ + *η*_*k*_, for *k* = 1,2,…, *p*, where *ϕ*_1_, *ϕ*_2_, *f*_*k*,1_, *f*_*k*,2_, *η*_*k*_ ~ *N*(0, **I**), and *p* = 1000, *n* = 200.

Moreover, we created sparse regression vectors by setting *β*_*k*_ = 0 for all *k* = 1,…, *p*, except for a small variable set *S*. For all *k* ∈ *S*, we chose the coefficient *β*_*k*_ independently and uniformly in [0,1] and let the size *s* = |*S*| varying between 4 and 10. Here, we employed the method used in Section 3.1 to achieve the censoring rates 0% and 20%. Then, a Cox model was constructed by (8).

To compare the power of StabSel and lasso to ranking variables, we adopted the strategy utilized by [19], that is, focusing on the probability that *γs* variables in *S* can be recovered correctly, where *γ* ∈ {0.1,0.3}. For lasso, this means that there is a regularization parameter such that at least ⌈*γs*⌉ variables in *S* are selected while all variables in *N* = {1,…, *p*}∖*S* are not selected. For stability selection, it stands for the fact that ⌈*γs*⌉ variables with highest selection frequency are all in *S*. In this example, we fixed the threshold value *π*_thr_ = 0.6 and *q*_Λ_ = ⌈(0.8*p*)^1/2^⌉ to determine a proper value for *λ*_min_.

**Figure 1:**
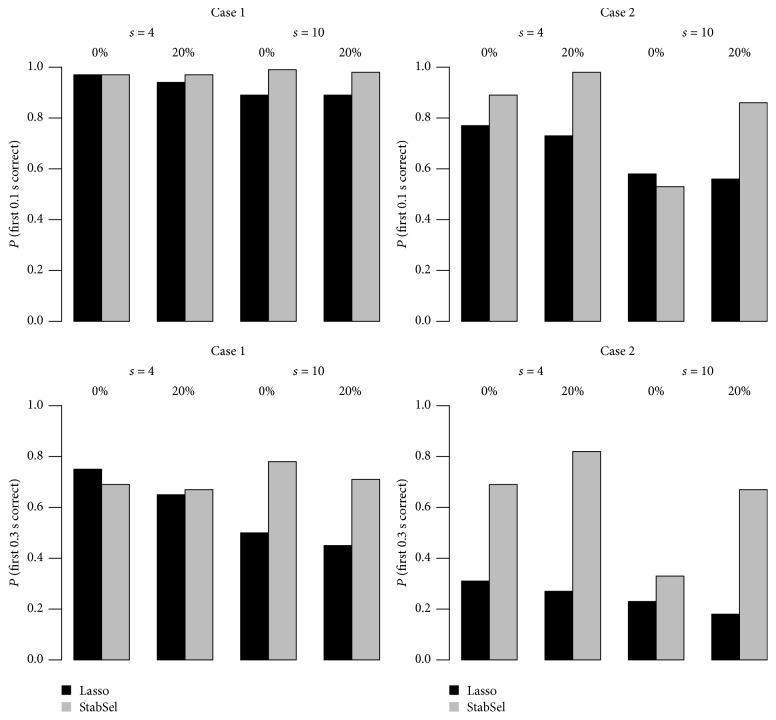
Selection probabilities of StabSel and lasso.

The top two subplots in Figure 1 correspond to the situation of *γ* = 0.1 while the bottom two subplots illustrate the results for *γ* = 0.3. Notice that the latter task is more challenging than the former one. When the covariates are independent in Case 1, lasso performs satisfactorily and the advantage of StabSel is not significant. In Case 2, the dominance of StabSel over lasso to identify important variables more correctly can be clearly seen, especially when faced with censored data. In the more challenging task in which more important variables are required to be ranked ahead (i.e., *γ* = 0.3), the superiority of StabSel is more significant. In conclusion, this experiment shows that StabSel is indeed helpful to enhance the ranking ability of lasso.

### 3.3. Simulation 3: Performance Comparison with Several Other Methods

Finally, we considered a simulated dataset used in [20]. There are *n* = 80 observations and *p* = 20 predictor variables. Each predictor was generated according to

(9) $$\begin{array}{c} x_{j}=z+\epsilon_{j},\;j=1,2,\ldots ,20,\;\;\epsilon_{j},z\overset{iid}{\sim }N_{80}\left(0,I\right). \end{array}$$

The response vector **y** was generated from an exponential distribution with hazard function

(10) $$\begin{array}{c} h_{i}\left(\;t\;\right)=h_{0}\left(\;t\;\right)\exp\left(\;0.5x_{i,5}+x_{i,10}+1.5x_{i,15}\;\right). \end{array}$$

As for the variables other than **x**_5_, **x**_10_, **x**_15_, the coefficient is zero. Altogether, three simulation studies were conducted with censoring rates 0%, 20%, and 40%, respectively. For StabSel, we fixed *π*_thr_ = 0.6 and *q*_Λ_ = ⌈(1.6*p*)^1/2^⌉. As mentioned in Section 2, the number of variables that lasso selects in each trial should be at least larger than the number of truly important variables. Thus, we increased the factor multiplying *p* in *q*_Λ_ because *p* is small in this simulation. We compared it with traditional stepwise search as well as some VSE techniques including BSS [20], PGA [18], RSMA [24], and ST2E [22]. The parameters involved in these methods were set according to the related literature.

Table 2 summarizes the selection frequencies of IV and UIV for each approach. The results demonstrate that although PGA performs better to exclude unimportant variables, it may miss some truly important variables. On the other hand, RSMA, ST2E, and StabSel can identify almost the same number of important variables; the difference only lies in the exclusion of unimportant ones. In this aspect, StabSel is observed to behave the best. As for BSS, its ability to guard against noise variables seems to be worse than the others although it works well to identify IVs.

**Figure 2:**
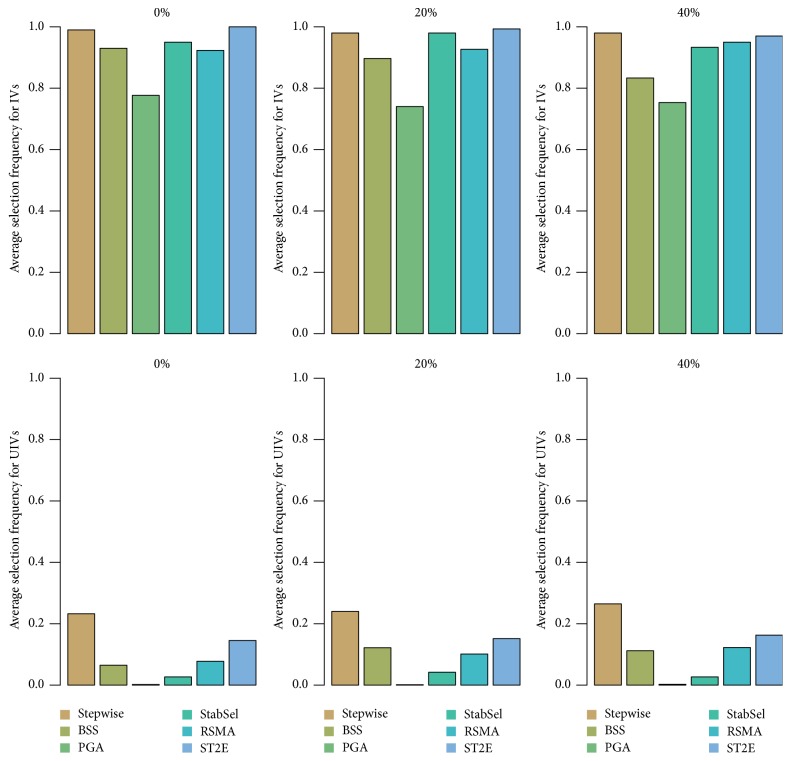
Average selection rate for different ensemble approaches.

In order to see more clearly the differences among the considered approaches, we computed the average* selection rate* of IV and UIV. For IV, it was computed as the selection probabilities averaged over all important variables. The metric was similarly estimated for UIV. The results are illustrated in Figure 2. The top three subplots are IVs while the bottom three ones are for UIVs. From Figure 2, we can come to some conclusions similar to those drawn from Table 2.

At the same time, we also utilized several other metrics to extensively evaluate each method. First, we computed the* selection success rate* [13]. Given an algorithm, it refers to the fraction of times among 100 runs that the algorithm correctly identifies the true model (i.e., the model only includes {**x**_5_, **x**_10_, **x**_15_}). Second, the* true positive rate* (TPR) and* true negative rate* (TNR) of each method were considered. In particular, TPR and TNR are as follows:

(11) TPR=1100·IV∑t=1100∑j∈IVIβ^j,t≠0,TNR=1100·UIV∑t=1100∑j∈UIVIβ^j,t=0,

where $\hat{\beta }={\left({\hat{\beta }}_{1,t},{\hat{\beta }}_{2,t},\ldots ,{\hat{\beta }}_{p,t}\right)}^{T}$ is the estimated coefficient vector in the *t*th simulation. In addition, |IV| and |UIV| represent the size of IV and UIV, respectively. The method “Oracle” corresponds to fitting a Cox model with only variables **x**_5_, **x**_10_, and **x**_15_. Usually, a good variable selection method should produce results as close as possible to those of Oracle.

It can be seen from Table 3 that stepwise method is hopeless to select variables since it can hardly find the true model. Among the VSE algorithms, StabSel always reaches the largest selection success rate, especially when the censoring rate is high. On the other hand, StabSel tends to achieve a model size closest to that of Oracle. As far as the prediction performance is concerned, StabSel almost always outperforms the other approaches.

## 4. Real-World Applications

In this section, we applied the compared VSE techniques to three real-world datasets, that is, PBC [31], Lung [32], and Rats [33]. These real datasets were taken from the R package survival. For the original PBC and lung sets, we simply ignored the observations containing missing data. In these situations, there are no means to know which variables are truly important or not. Aiming at evaluating the selection behavior of each method, we took the original variables as truly important ones (i.e., IVs). Then, some irrelevant variables were artificially added to these sets by following the strategy used in [25, 34]. These irrelevant variables were generated from a uniform distribution on the interval [0,1].  Table 4 lists the main characteristics of the used three datasets.

Analogous to the situation of simulation studies, the ensemble size was set as *B* = 200. The parameters involved in each method were set similarly to those used in simulations. For each dataset, the experiment was repeated 100 times. In each replication, a training set was randomly drawn from the given set with size being specified in Table 4. The rest of observations was then used as a test set to evaluate the prediction performance measured with C-index [35] (i.e., concordance index). In particular, we applied each algorithm to the training set to perform variable selection. Based on the selected variables, the parameters in the corresponding model were estimated and the C-index was estimated on the test set.  Table 5 shows the results obtained with each algorithm.

In terms of selection rate, it can be observed from Table 5 that BSS performs well to identify IVs. Nevertheless, it behaves worse to exclude UIVs. On the contrary, PGA shows the lowest selected rate of UIVs while it has the lowest selected rate of IVs. Therefore, BSS and PGA are not ideal selection methods. For the remaining methods, StabSel, RSMA, and ST2E behave similarly in identifying IVs. But when compared with StabSel, RSMA and ST2E include more irrelevant variables. In conclusion, StabSel achieves better performance on variable selection when being evaluated with selection rate.

Furthermore, the results of C-index in Table 5 reveal that the prediction performance of StabSel is competitive although it is not the best one. Furthermore, almost all ensemble methods tend to have low TPR values in these three real datasets. This is largely due to the fact that we directly consider all the original covariates as IVs among which some are actually uninformative.

## 5. Conclusions

As an ensemble method, StabSel [19] is the marriage of subsampling with a variable selection algorithm such as lasso. Due to its property of controlling false discovery rate, StabSel has a flexible manner to choose a proper amount of regularization. Another superiority of StabSel over lasso is that it requires less assumptions to achieve variable selection consistency. In this article, we extended StabSel to the Cox model. The specification of *λ*_min_ significantly affects the performance of StabSel since it controls the balance between false positive rate and false negative rate. We provide an explicit way to set a proper value for *λ*_min_ in the situation of Cox models. In comparison with other VSE techniques including PGA, BSS, RSMA, and ST2E, StabSel exhibits better selection ability to correctly identify important variables in a high-dimensional Cox model. At the same time, StabSel has satisfactory prediction performance. When the censoring rate is high, its advantage is even more significant. Therefore, StabSel can be considered as an alternative to explore the relationship between covariates and survival times in survival analysis.

## Figures and Tables

**Algorithm 1:**
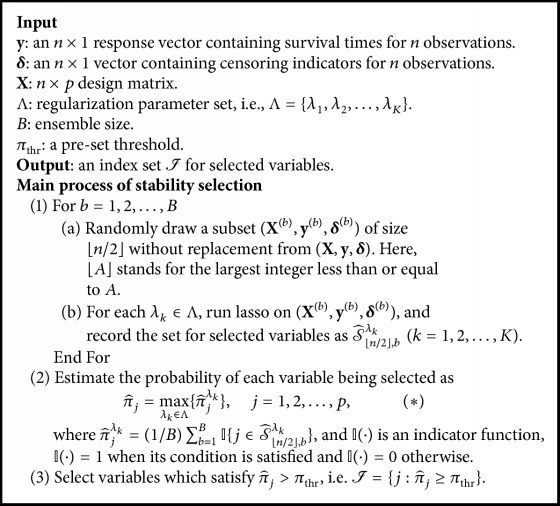
The stability selection algorithm for the Cox model.

**Table 1:** Selection frequencies of StabSel to identify IV and UIV.

	*x*_*j*_ ∈ IV			*x*_*j*_ ∈ UIV		
	(*j* = 1,2, 5)			(*j* = 3,4, 6,7, 8)		
	Min	Med	Max	Min	Med	Max
0%* censoring*						
*λ*_min_ = 0.3	67	69	73	0	0	1
*λ*_min_ = 0.2	75	77	81	0	1	3
*λ*_min_ = 0.1	77	81	84	3	7	15
20%* censoring*						
*λ*_min_ = 0.3	85	88	91	0	0	3
*λ*_min_ = 0.2	93	99	100	1	3	6
*λ*_min_ = 0.1	100	100	100	3	8	20
40%* censoring*						
*λ*_min_ = 0.3	49	76	98	0	0	2
*λ*_min_ = 0.2	94	98	100	0	1	6
*λ*_min_ = 0.1	100	100	100	3	6	14

**Table 2:** Selection frequencies of each method in Simulation 3.

Method	*x*_*j*_ ∈ IV			*x*_*j*_ ∈ UIV		
	Min	Med	Max	Min	Med	Max
0%* censoring*						
Stepwise	97	100	100	13	22	30
BSS	79	100	100	3	7	10
PGA	40	93	100	0	0	1
StabSel	91	97	97	0	3	5
RSMA	79	98	100	4	8	13
ST2E	100	100	100	10	15	18
20%* censoring*						
Stepwise	94	100	100	19	24	31
BSS	70	100	100	6	12	17
PGA	29	94	100	0	0	1
StabSel	94	96	97	1	3	5
RSMA	80	98	100	4	9	17
ST2E	94	100	100	8	15	23
40%* censoring*						
Stepwise	94	100	100	22	26	38
BSS	65	89	96	8	11	15
PGA	31	95	100	0	0	1
StabSel	97	100	100	1	3	7
RSMA	80	99	100	7	13	18
ST2E	91	100	100	11	15	25

**Table 3:** Results for each method in Simulation 3.

Method	Succ. rate	Size	TNR	TPR
0%* censoring*				
Stepwise	0.02	6.92	0.768	0.990
BSS	0.51	3.89	0.935	0.930
PGA	0.37	2.36	0.998	0.777
StabSel	0.55	3.30	0.973	0.950
RSMA	0.21	4.09	0.922	0.923
ST2E	0.01	5.47	0.855	1.000
20%* censoring*				
Stepwise	0.04	7.02	0.760	0.980
BSS	0.31	4.76	0.879	0.900
PGA	0.28	2.25	0.999	0.743
StabSel	0.57	3.33	0.914	0.957
RSMA	0.14	4.50	0.899	0.927
ST2E	0.05	5.55	0.849	0.993
40%* censoring*				
Stepwise	0.02	7.44	0.735	0.980
BSS	0.15	4.55	0.878	0.823
PGA	0.30	2.30	0.998	0.753
StabSel	0.61	3.51	0.968	0.990
RSMA	0.06	4.87	0.878	0.930
ST2E	0.03	5.68	0.837	0.970
Oracle	1.00	3.00	1.00	1.00

**Table 4:** Main characteristics of the used real-world datasets.

Dataset	Number of variables	Number of samples	Training size
PBC	15 (original covariates)	276	200
	+20 (random uniform)		
Lung	8 (original covariates)	167	100
	+20 (random uniform)		
Rats	3 (original covariates)	300	250
	+20 (random uniform)		

**Table 5:** The performance of each method on three real datasets.

Dataset	Metric	PGA	BSS	StabSel	RSMA	ST2E
PBC	Sel. rate					
	IVs (1–15)	0.299	0.597	0.518	0.543	0.605
	UIVs (26–35)	0.015	0.291	0.053	0.074	0.120
	C-index	0.792	0.812	0.819	0.826	0.835
	TPR	0.24	0.50	0.42	0.54	0.63
	TNR	0.98	0.60	0.99	0.96	0.96

Lung	Sel. rate					
	IVs (1–8)	0.284	0.607	0.477	0.476	0.466
	UIVs (9–28)	0.077	0.426	0.097	0.289	0.200
	C-index	0.631	0.703	0.695	0.680	0.695
	TPR	0.27	0.61	0.41	0.51	0.54
	TNR	0.92	0.55	0.83	0.71	0.74

Rats	Sel. rate					
	IVs (1–3)	0.627	0.890	0.850	0.893	0.997
	UIVs (4–23)	0.043	0.332	0.101	0.160	0.159
	C-index	0.800	0.870	0.853	0.869	0.693
	TPR	0.60	0.89	0.70	0.89	1.00
	TNR	0.91	0.67	0.90	0.84	0.84
